# Supplementary material for: Correction: Diversity of blaCTX-M-1-carrying plasmids recovered from Escherichia coli isolated from Canadian domestic animals
Source: PLoS One. 2024 Apr 9;19(4):e0302193. doi: 10.1371/journal.pone.0302193 (PMC11003620; doi:10.1371/journal.pone.0302193)
Supplement: S1 File — (PDF) [file pone.0302193.s001.pdf]

PLOS ONE

RESEARCH ARTICLE

Diversity of *bla*$_{CTX-M-1}$-carrying plasmids recovered from *Escherichia coli* isolated from Canadian domestic animals

Ashley C. Cormier[1], Gabhan Chalmers[1], Roxana Zamudio[2], Michael R. Mulvey[3], Alison E. Mather[2,4], Patrick Boerlin[1]*

1 Department of Pathobiology, University of Guelph, Guelph, Ontario, Canada, **2** Quadram Institute Bioscience, Norwich Research Park, Norwich, Norfolk, United Kingdom, **3** National Microbiology Laboratory, Public Health Agency of Canada, Winnipeg, Manitoba, Canada, **4** University of East Anglia, Norwich, Norfolk, United Kingdom

* pboerlin@uoguelph.ca

Check for updates

Abstract

Conserved IncI1 and IncHI1 plasmids carrying *bla*$_{CTX-M-1}$ have been found circulating in chickens and horses from continental Europe, respectively. In Canada, *bla*$_{CTX-M-1}$ is overwhelmingly the most common *bla*$_{CTX-M}$ variant found in *Escherichia coli* from chicken and horses and can be recovered at lower frequencies in swine, cattle, and dogs. Whole-genome sequencing has identified a large genetic diversity of isolates carrying this variant, warranting further investigations into the plasmids carrying this gene. Therefore, the objective of this study was to describe the genetic profiles of *bla*$_{CTX-M-1}$ plasmids circulating in *E. coli* from Canadian domestic animals and compare them to those recovered in animals in Europe. Fifty-one *bla*$_{CTX-M-1}$ positive *E. coli* isolates from chicken (n = 14), horses (racetrack horses n = 11; community horses n = 3), swine (n = 7), turkey (n = 6), dogs (n = 5), beef cattle (n = 3), and dairy cattle (n = 2) were selected for plasmid characterization. Sequences were obtained through both Illumina and Oxford Nanopore technologies. Genomes were assembled using either Unicycler hybrid assembly or Flye with polishing performed using Pilon. *bla*$_{CTX-M-1}$ was found residing on a plasmid in 45 isolates and chromosomally located in six isolates. A conserved IncI1/ST3 plasmid was identified among chicken (n = 12), turkey (n = 4), swine (n = 6), dog (n = 2), and beef cattle (n = 2) isolates. When compared against publicly available data, these plasmids showed a high degree of similarity to those identified in isolates from poultry and swine in Europe. These results suggest that an epidemic IncI1/ST3 plasmid similar to the one found in Europe is contributing to the spread of *bla*$_{CTX-M-1}$ in Canada. A conserved IncHI1/FIA(HI1)/ST2 plasmid was also recovered from nearly all racetrack horse isolates (n = 10). Although IncHI1/ST2 plasmids have been reported among European horse isolates, IncHI1/ST9 plasmids appear to be more widespread. Further studies are necessary to understand the factors contributing to these plasmids' success in their respective populations.

OPEN ACCESS

Citation: Cormier AC, Chalmers G, Zamudio R, Mulvey MR, Mather AE, Boerlin P (2022) Diversity of *bla*$_{CTX-M-1}$-carrying plasmids recovered from *Escherichia coli* isolated from Canadian domestic animals. PLoS ONE 17(3): e0264439. https://doi.org/10.1371/journal.pone.0264439

Editor: Axel Cloeckaert, Institut National de la Recherche Agronomique, FRANCE

Received: June 9, 2021

Accepted: February 10, 2022

Published: March 16, 2022

Copyright: © 2022 Cormier et al. This is an open access article distributed under the terms of the Creative Commons Attribution License, which permits unrestricted use, distribution, and reproduction in any medium, provided the original author and source are credited.

Data Availability Statement: **PA at Accept: Please check data access** Data will be available through Genbank once accepted (Bioproject number PRJNA777610).

Funding: This study was funded by the Natural Sciences and Engineering Research Council of Canada discovery grant (PB; 2015-03962; https://www.nserc-crsng.gc.ca/professors-professeurs/grants-subs/dgigp-psigp_eng.asp), the Canadian Institutes of Health Research (PB; CFC-150770; https://cihr-irsc.gc.ca/e/193.html) and the Medical

Research Council (AEM; MR/R000948/1; https://mrc.ukri.org/). The funders had no role in study design, data collection and analysis, decision to publish, or preparation of the manuscript.

Competing interests: The authors have declared that no competing interests exist.

Introduction

Since the early 2000s, the Canadian Integrated Program for Antimicrobial Resistance Surveillance (CIPARS), has reported extended-spectrum cephalosporin (ESC) resistance among *Escherichia coli* from beef cattle, swine, chicken, and turkey [1–4]. Other studies have also demonstrated ESC resistance among *E. coli* from horses [5, 6] and dogs [7] in Canada. When available, genotypic analysis of ESC-resistant *E. coli* from these animal species has identified bla_{CMY-2} as the major ESC resistance determinant and has documented the emergence of the bla_{CTX-M} family in Canada [7–12].

After having been detected globally for several years [13–17], bla_{CTX-M} variants were first detected in humans and companion animals in Canada in the early 2000s [18–20]. The wide dispersion and success of these resistance genes have been attributed to their association with epidemic strains and with mobile genetic elements (MGEs) such as plasmids, integrons, transposons, and insertion sequences [21]. These MGEs have allowed bla_{CTX-M} to move extensively within and between bacteria, and have provided the tools necessary for persistence in the absence of β-lactams through the co-location of multiple resistance determinants and co-selection by other antibiotics [22, 23].

Following bla_{CTX-M}'s emergence in Canada, variants $bla_{CTX-M-14,\ -15,\ -27,}$ and $bla_{CTX-M-55}$ have become those most frequently identified among *Enterobacterales* from both humans and animal species [24, 25]. This contrasts with $bla_{CTX-M-1}$, which is rarely identified in humans in Canada [25] but widespread in bacteria from pigs, cattle, and dogs and overwhelmingly the most common variant in bacteria from chicken and horses [6, 24]. This also contrasts with what has been reported in Europe where $bla_{CTX-M-1}$ has been regularly found among human isolates [26–29] and is the most common variant recovered from various production animals [30, 31]. Studies in Europe and Canada have demonstrated a large genetic diversity among isolates carrying $bla_{CTX-M-1}$, suggesting that clonal spread may not be the major or only driver in the spread of this variant [24, 30, 32, 33].

Using a variety of molecular techniques, researchers have identified related incompatibility (Inc) group HI1 plasmids circulating within European horse populations [34, 35]. Similarly, related IncI1 plasmids were observed in European chicken populations [32, 36], and in various food animal samples [29, 30]. IncI1 plasmids have been well characterized and are frequently found to carry antimicrobial resistance determinants, apart from $bla_{CTX-M-1}$ [37]. These plasmids have four conserved regions, encoding replication (e.g., *inc*, *repY*, *repZ*), stability (e.g., *parA*, *parB*), leading (e.g., *ardA*, *psiA*) and conjugative transfer (e.g., *traA-Y*) [37].

The appearance of conserved IncI1 and IncHI1 plasmids across Europe raises two questions: First, are there also conserved $bla_{CTX-M-1}$-carrying plasmids circulating in bacteria from domestic animals in Canada? Second, if this is true, are they the same as those recently recovered across Europe? Therefore, the objective of this study was to investigate and describe the genetic profiles of $bla_{CTX-M-1}$ plasmids circulating in bacteria from Canadian domestic animals. For this purpose, we used the Illumina and Oxford Nanopore platforms and hybrid assembly approaches to assemble and subsequently compare $bla_{CTX-M-1}$ plasmid sequences of recent *E. coli* isolates from chicken, turkeys, pigs, dairy cattle, beef cattle, horses, and dogs from Canada.

Materials and methods

Isolate selection

Fifty-one $bla_{CTX-M-1}$-positive *E. coli* isolates from various commodities and sources were selected for this study (Table 1). Isolates from chicken, turkey, swine, beef cattle, racetrack

Table 1. Sampling characteristics of *E. coli* isolates selected for analyses.

Animal Species	Number of isolates	Sampling location	Sample type	Sample year(s)	Reference
**Chicken**	14	Ontario	cecal	2015–2016	[12]
**Turkey**	6	Canada	fecal	2016–2017	[11]
**Beef cattle**	3	Alberta	fecal	2014–2015	[8]
**Dairy cattle**	2	Ontario	fecal	2017	This study
*Horse	11	Ontario (racetrack)	fecal	2017	[24]
	3	Ontario (OVC)	fecal	2018	This study
**Swine**	7	Ontario	cecal	2015–2016	[12]
**Dog**	5	Ontario	fecal	2016	[7]
**Total**	51				

* Horse isolates were collected from two different studies conducted in Ontario, at the Ontario Veterinary College (OVC) large animal clinic and at a racetrack.

https://doi.org/10.1371/journal.pone.0264439.t001

horses, and dogs were selected from previous studies based upon the detection of $bla_{CTX\text{-}M\text{-}1}$. The Ontario Veterinary College (OVC) horse and dairy cattle isolates were from a larger collection of $bla_{CTX\text{-}M}$ group-1 positive isolates collected by the Dr. J. Scott Weese laboratory, OVC. Briefly, samples of 200 mg of feces taken from horses entering the OVC large animal clinic and dairy cattle from a single farm in Ontario, Canada were incubated for 18–24 hrs at 37˚C in LB broth (Becton, Dickinson and Company, Sparks, MD, USA) (9 ml) without antibiotics. The resulting cultures were plated (10 μl) on CHROMID® ESBL agar (BioMérieux, Laval, QC, Canada) and incubated for 18–24 hrs at 37˚C. Putative *E. coli* isolates were frozen for later use. Susceptibility testing was used to confirm the extended-spectrum beta-lactamase (ESBL) producers' phenotype in accordance with the guidelines of the Clinical Laboratory Standards Institute (CLSI) [38].

All or up to a maximum of 14 $bla_{CTX\text{-}M\text{-}1}$-positive isolates were selected from each animal species source. In instances where more than 14 isolates were available from an animal species, isolates were selected using a random number generator. If several isolates were available from the same sample, a random number generator was also used to select one isolate from this sample.

Whole genome sequencing and assembly

DNA extractions for short- and long-read sequencing were performed using the Lucigen MasterPure DNA Purification kit with complete removal of RNA, according to manufacturer's instructions (Lucigen, Middleton, WI, USA). Short-read sequences were obtained using MiSeq (PE300), HiSeq (PE100), or NextSeq (PE150) technology (Illumina, San Diego, CA, USA) after library preparation using Nextera XT kits (Illumina). Sequencing was performed at the Advanced Analysis Centre, University of Guelph, ON, Canada; McGill University and Génome Québec Innovation Centre, McGill University, QC, Canada; and at the National Microbiology Laboratory of Canada, MB, Canada. Long-read genome sequencing and assembly were performed on all isolates except those from turkey since these were performed in a previous study [11]. Long-read sequences were obtained in-house using the Oxford Nanopore MinION (Oxford Nanopore Technologies, Oxford, UK) with FLO-MIN106D flow cells, following library preparation using the Ligation Sequencing Kit (SQK-LSK109), Native Barcoding Kit (EXP-NDB104 and EXP-NBD114) as per manufacturer instructions.

Basecalling and demultiplexing were performed using MinKNOW v1.4.2 (Oxford Nanopore Technologies), and Porechop v.0.2.4, respectively [39], or Guppy Basecaller v3.3 (Oxford Nanopore Technologies). Genomes were assembled using Unicycler v0.4.4 hybrid assembly

[40] and Flye v2.6 [41] with five rounds of Pilon polishing v1.25 [42]. Pilon was used to correct potential errors in the long-reads using short-reads. Polished Flye assemblies were manually circularized by mapping short- and long-reads against the original circularized Flye assembly to determine the correct sequence of nucleotides necessary to close the plasmid. Frequently, both polished Flye and Unicycler outputs yielded similar results for each plasmid of interest. To determine which assembly would be used in the final analyses, a series of quality control measures were taken. First, ARIBA v2.14.1 [43] and ABRicate v0.9.8 [44, 45] were used to confirm that genes were not lost in assembly compared to short-read assemblies. Socru v2.2.4 [46] was used to identify large-scale misassemblies in the entire genome by the arrangement of ribosomal operons. Long and short reads were then mapped to the final plasmid assemblies to determine if there were any noticeable gaps in coverage that may indicate a misassembly. Finally, if both assemblies remained comparable in quality until this point, Snippy v4.4.5 [47] was used to determine the number of short nucleotide variations (SNVs) between the short-reads and the long-read/hybrid plasmid assemblies. The plasmid assembly with fewer SNVs was selected for further analyses.

Following genome assembly, Achtman sequence types were confirmed using PubMLST (https://pubmlst.org/), phylotypes and serotypes were determined using EZClermont [48, 49] and ECTyper [50], respectively.

Plasmid alignment and gene annotation

The resulting $bla_{CTX-M-1}$ plasmids were compared using Easyfig [51] to identify conserved plasmids. Comparisons were made between plasmids recovered in Canada, continental Europe and with the IncI1 plasmid, R64 (Bioproject: PRJNA224116). Gene identification was performed using ResFinder v3.1.0 [45], PlasmidFinder v2.0.1 [52], and ISE Scan [53]. Plasmid multilocus sequence typing (pMLST) was performed with PubMLST (https://pubmlst.org/). When necessary, mapping of short-reads was used to confirm STs. Reference mapping was also used to look for the presence of the *fos* operon, as well as for the *pemK*, *ccdA/B*, *relE/B*, *parD/E*, *vagC/D*, *hok/sok*, *pndA/C*, and *srnB/C* addiction systems on all ESC resistance plasmids.

Validation of predicted antimicrobial susceptibilities

To validate the predicted antimicrobial susceptibilities of the $bla_{CTX-M-1}$-carrying plasmids characterized in this study, a subset of 14 plasmids were assessed by antimicrobial susceptibility testing (AST) using the disk diffusion method. A single plasmid from each resistance profile within each Inc group (S1 Table) was chosen at random for transformation and AST. Plasmid preparations were made using the QIAGEN® Plasmid Purification Mini Kit, as per manufacturer instructions (QIAGEN, Valencia, CA, USA). Plasmids were transformed via electroporation into *E. coli* ElectroMAX™ DH10B™ (Invitrogen by Thermo Fisher Scientific, Carlsbad, CA, USA) and transconjugants selected on LB agar containing 1 mg/L ceftriaxone (Sigma-Aldrich, St. Louis, MO, USA). Polymerase chain reaction amplification of bla_{CTX-M} [54], plasmid preparations, and gel electrophoresis were performed on transformants to confirm the presence of a single $bla_{CTX-M-1}$-carrying plasmid. Once confirmed, AST was performed according to the CLSI guidelines [38] using the following antimicrobials: cefotaxime, trimethoprim, spectinomycin, tetracycline, chloramphenicol, ciprofloxacin, gentamicin, kanamycin (BD, Sparks, MD, USA), and sulfonamides (Oxoid Ltd, Hampshire UK). For this study, those isolates that fell into the intermediate zone of inhibition were considered resistant. Susceptibility testing for streptomycin was not performed due to the presence of natural resistance to this antimicrobial in *E. coli* DH10B.

SNP analyses of IncI1/ST3 and IncHI1 plasmids

A single representative plasmid (pAC112.1 and pAC1185-1-1) was selected from the IncI1/ST3 and IncHI1/ST2 plasmid groups based on assembly quality; those with the highest depth of coverage and the fewest SNVs were selected. Using these plasmids, a selection of publicly available plasmids with the same pMLST and/or Inc-type were identified by the Nucleotide Basic Local Alignment Search Tool (BLASTN) [55], available through NCBI. These included five IncI1/ST3 plasmids from various food animals, as well as one IncHI1/ST2 and eight IncHI1/ST9 plasmids from horses (Table 2); all plasmids carried *bla*$_{CTX-M-1}$.

Prokka v1.14.6 [59] was first used to annotate the plasmid sequences of interest. Roary v3.13.0 [60] was then used on IncHI1 and IncI1/ST3 plasmids separately, to identify core and accessory genes among international and Canadian plasmids. For this analysis, core genes were defined as those present in 80% of plasmids with a minimum identity of 95%. Pairwise comparison of single nucleotide polymorphisms (SNPs) was then performed through snp-dist v0.7.0 [61].

Results

Assemblies, incompatibility groups and pMLST

A combination of Unicycler and polished Flye assemblies was selected for analysis. Hybrid assembly showed that *bla*$_{CTX-M-1}$ was located on 45 plasmids and on six occasions on chromosomes. Chromosomal *bla*$_{CTX-M-1}$ was identified in isolates from all animal species. Plasmid were recovered from 27 different Achtman STs.

PlasmidFinder identified six Inc types among the plasmids. Two Inc-type markers were identified on 13/45 plasmids (S1 Table). Plasmids with multiple Inc types all carried at least one IncF marker. The most common Inc-type reported was IncI1 (n = 28), with plasmids originating from chicken (n = 12), turkey (n = 5), swine (n = 6), beef cattle (n = 2), and dog isolates (n = 3). The second most common Inc-type was from a group of plasmids identified as IncHI1/FIA(HI1) (n = 11); all but one of the plasmids from this group were recovered from racetrack horse isolates. The remaining plasmid from this group was from a dog isolate. Four IncN plasmids from cattle isolates (dairy and beef; n = 3) and an OVC horse, as well as two IncFIB/FII plasmids from horses (racetrack and OVC), were also recovered. Plasmids varied

Table 2. Publicly available plasmid sequences retrieved through NCBI BLASTN for phylogenetic comparison with plasmids recovered in this study.

Plasmid	Accession number	Inc-type	ST	Animal	Origin	Reference
**p14019095**	MK181557	I1	3	Chicken	Denmark	[56]
**pHV292**	KM377239	I1	3	Chicken	Switzerland	[33]
**pCOV33**	MG649046.1	I1	3	Chicken	France	[57]
**p15090172**	MK181562	I1	3	Swine	Denmark	[56]
**unnamed**	CP009580.1	I1	3	Swine	Netherlands	[58]
**p15S04714-1**	MT586601.1	HI1	9	Horse	Netherlands	[35]
**p15S04779-4**	MT586602.1	HI1	9	Horse	Netherlands	[35]
**p15S04829-4**	MT586603.1	HI1	9	Horse	Netherlands	[35]
**p97974-2T**	MT586605.1	HI1	9	Horse	Czech Republic	[35]
**p99063**	MT586606.1	HI1	9	Horse	Czech Republic	[35]
**p99783-3T**	MT586607.1	HI1	9	Horse	Czech Republic	[35]
**p99975-2**	MT586608.1	HI1	9	Horse	Czech Republic	[35]
**p100063-3**	MT586609.1	HI1	9	Horse	Czech Republic	[35]
**p10068**	MT586604.1	HI1	2	Horse	Czech Republic	[35]

https://doi.org/10.1371/journal.pone.0264439.t002

in size, however, remained relatively consistent within incompatibility groups (S1 Table). The median plasmid size for the major incompatibility types IncI1 and IncHI1 plasmids were 107,168bp and 213,856bp, respectively.

Plasmid multi-locus sequence typing identified ST3 as the most common (26/28) among IncI1 plasmids, ST2 (10/11) among IncHI1/FIA(HI1) plasmids, and ST1 among IncN plasmids (4/4).

Antimicrobial resistance genes and predicted susceptibility

For this study, multi-drug resistance plasmids were defined as having resistance determinants to three or more antimicrobial classes. A total of 41 out of 45 plasmids encoded multi-drug resistance (Table 3). Two plasmids carried determinants for two antimicrobial classes and four plasmids carried β-lactam resistance determinants only (i.e., $bla_{CTX-M-1}$). Predicted resistance phenotypes of 14 plasmids were successfully confirmed through AST as per CLSI guidelines (S1 Table). The most common resistance genes co-located on the same plasmid as $bla_{CTX-M-1}$ were to sulfonamides (n = 41) and tetracyclines (n = 32), followed by streptomycin (n = 18), spectinomycin (n = 17), trimethoprim (n = 15), gentamicin (n = 15), chloramphenicol (n = 11), and kanamycin (n = 4). Aminoglycoside resistance determinants were the most numerous on plasmids recovered from horse isolates, except for a single IncHI1/FIA(HI1)/ST9 plasmid from a dog isolate. Sulfonamide and/or tetracycline resistance determinants were identified on all but one IncI1 plasmid; 22/28 of these plasmids carried resistance determinants to both (S1 Table).

Identification of elements or genes associated with the persistence and mobilization of bla_{CTXM-1}

Following gene annotation of all plasmid assemblies, the *fos* operon was found only on a single IncHI1/FIA(HI1)/ST9 plasmid from a dog isolate. The *pndA/C* addiction system was present on all IncI1 plasmids and the *relE/B* addiction system was present on all IncHI1 plasmids. None of the addiction systems investigated were found on the IncN or IncFIB/FII plasmids.

The $bla_{CTX-M-1}$ gene was present downstream of the insertion sequence (IS) *ISEcp1* on all IncI1 plasmids (n = 28) and 4/6 chromosomes. In two of these chromosomes (Isolates 76-2-1 and 446–1), the epidemic IncI1/ST3 plasmid appears to have been integrated. The isolates

Table 3. The number of plasmids from each animal species with the corresponding predicted resistance phenotype.

*Animal Species (#)	**CTX	SUL	TET	STR	SPT	GEN	KAN	TMP	CHL
**D(1), Bc(1), Dc(2)**	R								
**S(1), T(1)**	R	R							
**Bc(2), S(3), D(2), C(11), T(3)**	R	R	R						
**S(2), C(1)**	R	R		R	R			R	
**H_{OVC}(2), H_{RT}(1)**	R	R		R	R	R		R	
**H_{RT}(1)**	R	R		R	R	R	R		R
**T(1)**	R	R	R	R	R	R	R		
**D(1)**	R	R	R	R		R	R		R
**H_{RT}(8)**	R	R	R	R	R	R		R	R
**H_{RT}(1)**	R	R	R	R	R	R	R	R	R
**Total plasmids with predicted phenotype**	45	41	32	18	17	15	4	15	11

* S-Swine; C-Chicken; T-Turkey; D-Dog; Bc-Beef cattle; Dc-Dairy cattle; H_{OVC}- OVC horse; H_{RT}-Racetrack horse

** CTX-Cefotaxime; SUL-Sulfonamide; TET-Tetracycline; STR-Streptomycin; SPT-Spectinomycin; GEN-Gentamicin; KAN-Kanamycin; TMP-Trimethoprim; CHL-Chloramphenicol.

https://doi.org/10.1371/journal.pone.0264439.t003

possessing chromosomally encoded $bla_{CTX-M-1}$ were each of a different Achtman ST and the insertion site was different for each one. On most IncI1 plasmids (n = 23) *ISEcp1* was the only insertion sequence upstream of $bla_{CTX-M-1}$ and was inserted within the shufflon region. However, in five plasmids, an additional IS element (i.e., IS1, IS4, or IS5) had inserted between $bla_{CTX-M-1}$ and *ISEcp1*, effectively truncating *ISEcp1* in four instances. In contrast, *ISEcp1* was not found on IncN, IncHI1/FIA(HI1), or IncFIB/FII plasmids. Instead, $bla_{CTX-M-1}$ was located in regions heavily populated with a variety of IS families (e.g. IS1, IS3, IS4, and IS6) and other resistance determinants on IncHI1/FIA(HI1) and IncFIB/FII plasmids. On IncN plasmids, $bla_{CTX-M-1}$ was surrounded by one to three insertion sequences from either the IS1, IS3, or IS6 families. The orientation of the IS in these regions was not always consistent and at times differed between plasmids of the same Inc-type.

Confirmation of conserved plasmids

Based on Easyfig alignments, conserved plasmids were found in multiple isolates from a single host species, as well as in isolates from several different animal species. These include conserved IncI1/ST3 plasmids (S1 Fig) among chicken (n = 12), turkey (n = 4), swine (n = 6), dogs (n = 2), and beef cattle (n = 2). Conserved IncHI1/ST2 plasmids (S2 Fig) were also identified among racetrack horses (n = 10), IncN/ST1 plasmids among a community horse isolate (n = 1), beef cattle (n = 1) and dairy cattle (n = 2), and IncFIB/FII plasmids in racetrack (n = 1) and community horse isolates (n = 1). Only the two major groups of conserved plasmids were explored further (i.e., IncI1/ST3 and IncHI1/FIA(HI1)/ST2).

When a random selection of IncI1/ST3 plasmids was compared to the prototype plasmid R64, an approximately 20kbp section of the R64 plasmid was missing among the IncI1 plasmid recovered in this study. The genes identified in this section include those encoding IS elements and various metal and drug resistances (i.e., arsenic, tetracyclines, and aminoglycosides; Fig 1). The chicken plasmid pAC1185-1-1 was selected as a representative for the IncI1/ST3 plasmid population and was processed through NCBI BLASTN® to search for similar plasmids. A selection of five IncI1/ST3 plasmids was retrieved for further comparison (Table 2). Three-hundred and seventy-one different genes were identified from the IncI1/ST3 plasmids recovered in this study and those listed in Table 2, out of which 89 were identified as core genes. The core genes represent approximately 63% of the median length of IncI1/ST3 plasmids recovered in this study. Pairwise SNP comparisons of core gene showed ten Canadian plasmids with less than 50 SNPs when compared to the five European plasmids listed in Table 2 (S2 Table). An additional seven Canadian plasmids had only 10–50 SNPs when compared against multiple European plasmids selected for this analysis. Plasmids with less than 50 SNPs when compared with European plasmids were from chicken, turkey, swine, beef cattle and dog isolates; five of which were recovered from *E. coli* ST10 and three from ST117 (S1 and S2 Tables).

Five hundred and twenty-one different genes were identified from the IncHI1 plasmids, out of which 216 were identified as core genes. The core genes represent approximately 77% of the median length of IncHI1 plasmids recovered in this study. When compared with representative plasmids from Europe (Table 2) using pairwise SNP analyses, European horse plasmid p10068 (ST2) was distinct from IncHI1/ST2 horse plasmids recovered in this study, whereas Canadian dog plasmid pAC48-1-1 showed few SNPs when compared to European horse plasmids of the same ST (S3 Table).

Discussion

A group of conserved IncI1/ST3 plasmids was found among a variety of poultry, swine, dog, and cattle isolates from Canada. When compared against assemblies available through NCBI,

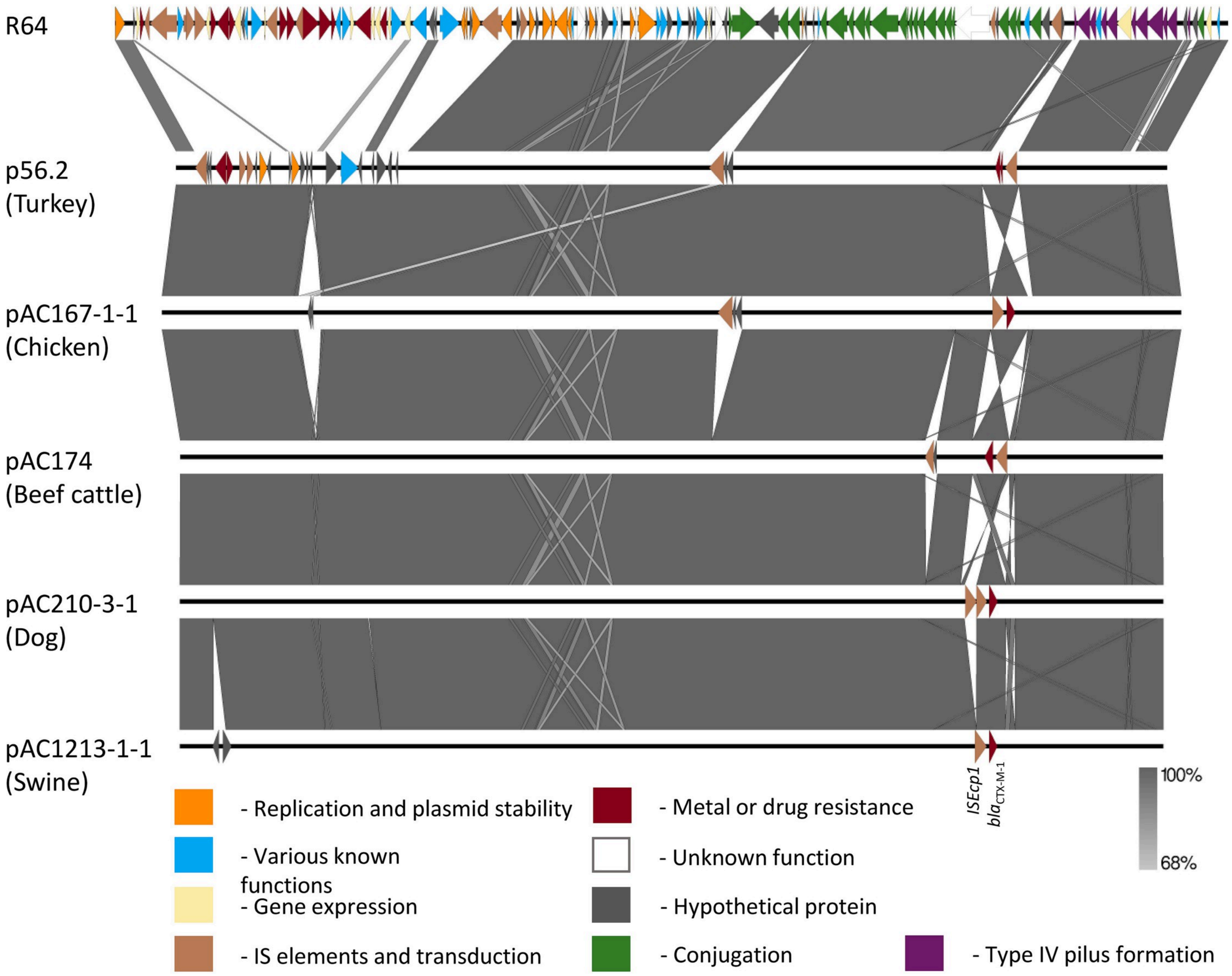

Fig 1. Easyfig alignment of representative IncI1/ST3 plasmids from chicken, turkey, swine, dogs and beef cattle, and R64.

https://doi.org/10.1371/journal.pone.0264439.g001

these plasmids showed a high structural similarity to those identified in isolates from poultry and swine in Europe. Core gene SNP analysis confirmed this similarity, therefore, providing evidence that bla_{CTXM-1} among *E. coli* from major production animals in Canada is being mobilized by the same epidemic plasmid previously identified in European isolates. Canadian plasmids exhibiting less than 50 SNPs (n = 17) when compared to plasmids of European origins were recovered from a diversity of animals and *E. coli* STs. However, 47% of plasmids were recovered from either *E. coli* ST10 or ST117. These results suggest that a widespread epidemic IncI1/ST3 plasmid is associated with the global dissemination of bla_{CTXM-1}, but that it's spread by conjugation is also supported by the expansion of highly successful bacterial clones.

The IncI1 plasmids from this study were, in general, multidrug resistance plasmids (median resistance to two classes of antimicrobial in addition to β-lactams). Sulfonamide and/or tetracycline resistance determinants were identified on almost all conserved IncI1/ST3 plasmids. Fortunately, critically important resistances (e.g. to aminoglycosides) were rarely encoded on

these plasmids. In recent years, the Canadian government and production animal industries have taken steps to limit the use of medically important antimicrobials for preventive medicine [62, 63]. However, based upon the most recently available data as of 2018, tetracyclines and sulfonamides remained some of the most heavily used antimicrobials in food animals in Canada [1]. Therefore, the continued use of these less important antimicrobials has the potential to reduce the efficacy of restrictions on the use of critically important antimicrobials such as ESCs through co-selection of these multi-drug resistant plasmids. These results warrant further *in vivo* investigations to assess whether the on-farm use of tetracyclines and/or sulfonamides does indeed influence the persistence of ESC resistance plasmids through co-selection.

The lower prevalence of *bla*$_{CTX-M-1}$ among *E. coli* from animal species other than chicken in Canada [24] may be indicative that this conserved IncI1 plasmid has first emerged in this species and is just starting to spread in other animal species, and in time may become more broadly and evenly distributed. It should also be noted that the chicken isolates used in this study were collected from samples acquired between 2015 and 2016, only one to two years after the cessation of ceftiofur use by the poultry industry [63]. The presence of this plasmid may have been driven by the previous use of ceftiofur in chicken, and subsequentially maintained by factors such as addiction systems (e.g. *pndA/C*), plasmid transmissibility, unknown metabolic advantages provided by the plasmid, or co-selection. Previously, *bla*$_{CMY-2}$ was the main resistance determinant responsible for ESC resistance in poultry populations in Canada [10, 12]. Infrequently, these plasmids carried resistance determinants to other classes of antibiotics when originating from poultry [64]. In contrast, the epidemic IncI1/ST3/CTX-M-1 plasmid recovered in this study encodes resistance to several antibiotics commonly used in Canada. As a result, over time, and if co-selection or other fitness factors are indeed acting upon these plasmids, *bla*$_{CTX-M-1}$ may become the major ESC resistance determinant among Canadian poultry. Further monitoring in animal populations and *in vivo* studies are warranted to test this hypothesis.

Consistent with what has been reported by Irrgang et al., 2018 [30], the *ISEcp1* mobile element encompassing the *bla*$_{CTX-M-1}$ was inserted between the PilV protein gene responsible for conjugation, and the shufflon-specific recombinase of our IncI1/ST3 plasmids. These authors demonstrated that *in vitro* conjugation was not interrupted by the insertion of the gene cassette in this region [30]. The broad genomic diversity of isolates carrying these plasmids in our study [24] suggest that this is the same under field conditions. The IncI1/ST3 plasmid is the most frequently recovered plasmid in this study and was always carrying *ISEcp1* in association with *bla*$_{CTX-M-1}$. The association of *bla*$_{CTX-M-1}$ with ISEcp1 and the IncI1 shufflon is not unique to this study and has been widely reported [32, 37]. The presence of this IS and plasmid-associated *bla*$_{CTX-M-1}$ on the chromosome of several of our isolates highlights again the important role that these mobile genetic elements play in the spread of *bla*$_{CTX-M-1}$, as with other CTX-M variants.

Conserved IncHI1/FIA(HI1)/ST2 plasmids were identified among all but one racetrack horse isolate from this study, but not in other horses from the local population served by the OVC large animal clinic which showed more diversity in plasmid type. These IncHI1/FIA (HI1)/ST2 plasmids were found in seven different *E. coli* STs, suggesting that clonal expansion was not the major driver in the spread of this plasmid within the racetrack. The racetrack sampled in this study frequently boards horses of international origins. In contrast, horses entering the OVC large animal clinic are more likely to be from the local Ontario community. This could explain the difference in the types of plasmids recovered from each source. The racetrack represents an environment with fewer opportunities for interference from outside sources when compared to community farms that may be more heavily influenced by other animals and humans. Highly similar IncHI1 plasmids are circulating in *E. coli* from horses in European

countries [34, 35]. Although the dominant plasmid sequence type detected in European horses has been ST9, ST2 has also been reported [35]. When comparing the plasmids recovered in this study to those identified in Europe, the racetrack horse plasmids (ST2) show a higher degree of genetic diversity than the European isolates (mainly ST9) within their core genes. Additionally, a representative IncHI1/ST2 plasmid (p10068) from the Czech Republic is no more or less similar to other IncHI1/ST2 plasmids recovered in this study than they are to ST9 plasmids from Europe. Additionally, the lack of diversity among IncHI1/ST9 plasmids in comparison to IncHI1/ST2 plasmids may suggest that IncHI1/ST9 plasmids may have emerged more recently than the IncHI1/ST2 plasmids.

Lastly, differences seen between the various horse populations explored (community vs. racetrack) may also be the result of the racetrack representing an environment with fewer opportunities for interference from environmental and human sources. It should be noted that the availability of community-derived horse samples was limited in comparison to those from the racetrack horses described in this study. Therefore, the results obtained here may not have fully captured the diversity of $bla_{CTX-M-1}$ plasmids circulating the Ontario horse population or the presence of a conserved plasmid population similar to the one found in the racetrack horses.

Many factors could be contributing to the dominance of this large IncHI1/FIA(HI1)/ST2 plasmid among racetrack horse isolates. First, these plasmids provide resistance to a large number of antibiotics. To our knowledge, there has been no data on antimicrobial use in horses in Canada reported recently. However, discussions with veterinary practitioners suggest that penicillin, gentamicin, and trimethoprim-sulfonamide combinations are among the first-line antimicrobials administered through the OVC (personal communication: Dr. Luis Arroyo Castro, OVC), falling in line with what had been reported in the early 2000s [65]. Frequent use of these antimicrobials would provide the opportunity for direct selection and co-selection of ESC resistance, and potentially support their persistence. Similar to the IncI1 plasmids recovered in this study, an addiction system was also found on the conserved IncHI1/FIA(HI1) plasmids. In the absence of antimicrobial use, this system would help to maintain this particularly large plasmid within the population. Lastly, these plasmids may provide a yet unknown additional fitness advantage for the bacterium, other than that afforded by the *fos* operon.

The *fos* operon involved in short-chain fructooligosaccharide metabolism is suspected to contribute to the success of the strains carrying IncHI1 plasmids in the equine intestinal tract [66]. Despite their presence in a variety of genetically unrelated isolates and widespread presence in the racetrack horses, none of the IncHI1/ST2 plasmids recovered in this study carried the *fos* operon. The single IncHI1/ST9 plasmid recovered from a dog isolate was the only one in this study to possess this carbon utilization system. These results mirror that of Valcek et al., 2021 [35] who found that IncHI/ST2 plasmids did not carry the *fos* operon, whereas IncHI1/ST9 plasmids did. Therefore, one can wonder whether there are additional characteristics of IncHI1 plasmids other than the *fos* operon that could also be contributing to their success in horse populations. Based on the findings from Valcek et al., 2021 [35] it is possible that the *fos* operon provides some advantage for IncHI1/ST9 plasmids, over IncHI1/ST2. However, IncHI1/ST9 plasmids may have yet to appear in the racetrack surveyed in this study, thereby limiting any comparisons that can be made between the success of IncHI1/ST9 vs. IncHI1/ST2 plasmids in this horse population.

Conclusions

Our observations show that similar to what has been observed in animals in Europe, a conserved epidemic IncI1/ST3 plasmid is predominantly responsible for the dissemination of $bla_{CTX-M-1}$ across a variety of animal species in Canada. More extensive studies comparing $bla_{CTX-M-1}$

plasmids from a diverse set of geographical regions are warranted to further clarify the relationships between international IncI1/ST3 plasmids and their transmission pathways. Due to the diversity of isolates often found carrying the conserved $bla_{CTX-M-1}$ plasmids, and based upon results from previous conjugation studies [67], we hypothesize that the success of $bla_{CTX-M-1}$ in chicken may, in part, be the result of enhanced conjugative abilities of these plasmids.

Isolates from racetrack horses carried mostly IncHI1/FIA(HI1) plasmids. Based on pairwise SNP comparisons this is likely the result of the intercontinental movement of horses. The $bla_{CTX-M-1}$ plasmids circulating in the bacteria from this racehorse population seem to differ from those found in the local Canadian horse population and also differ from the more conserved main ST9 plasmids found in European horses.

The dominant presence of two conserved $bla_{CTX-M-1}$ plasmids in chicken and racehorses, respectively, warrants further investigations on the mechanisms and plasmid characteristics that are contributing to the overwhelming success of these plasmids. The absence of the *fos* operon in the plasmids of this study suggests that additional factors and host adaptations may be involved in this success. Explanations for the lack of penetration of these plasmids in bacterial pathogens from humans in Canada are also needed.

Supporting information

S1 Fig. Easyfig alignment of IncI1/ST3/CTX-M-1 plasmids from *Escherichia coli* isolated from Canadian domestic animals.
(TIFF)

S2 Fig. Easyfig alignment of IncHI1/ST2/CTX-M-1 plasmids from *Escherichia coli* isolated from Canadian racetrack horses.
(TIFF)

S1 Table. Genotypic and phenotypic characteristics of $bla_{CTX-M-1}$ plasmids recovered from *Escherichia coli* isolated from Canadian domestic animals. * pMLST could not be confirmed, however the closest ST, based upon mapping short reads to the genes of interest, has been listed.
(XLSX)

S2 Table. A heatmap showing a pairwise SNP comparisons of IncI1/ST3/CTX-M-1 plasmids core genes from bacteria isolated from European and Canadian domestic animals. The numbers in the cells represent the number of SNP between the plasmids in comparison. Plasmid names are colour-coded by the animal species from which they were recovered.
(XLSX)

S3 Table. A heatmap showing a pairwise SNP comparisons of IncHI1/CTX-M-1 plasmids core genes from bacteria isolated from European and Canadian domestic animals. The numbers in the cells represent the number of SNP between the plasmids in comparison. Plasmid names are colour-coded by the animal species from which they were recovered.
(XLSX)

S1 Data.
(XLSX)

Acknowledgments

Many thanks to the members of Dr. Alison Mather's team for their guidance in genome assembly and analysis, and Dr. Scott Weese for the use of his $bla_{CTX-M-1}$ carrying isolates.

Author Contributions

Conceptualization: Patrick Boerlin.

Data curation: Ashley C. Cormier.

Formal analysis: Ashley C. Cormier.

Funding acquisition: Alison E. Mather, Patrick Boerlin.

Investigation: Ashley C. Cormier, Gabhan Chalmers.

Methodology: Ashley C. Cormier, Gabhan Chalmers, Roxana Zamudio, Alison E. Mather.

Project administration: Patrick Boerlin.

Resources: Michael R. Mulvey.

Supervision: Patrick Boerlin.

Writing – original draft: Ashley C. Cormier.

Writing – review & editing: Ashley C. Cormier, Gabhan Chalmers, Roxana Zamudio, Michael R. Mulvey, Alison E. Mather, Patrick Boerlin.

References

1. Government of Canada. 2018 Canadian Integrated Program for Antimicrobial Resistance Surveillance (CIPARS). 2020 Mar.
2. Boerlin P, Travis R, Gyles CL, Reid-Smith R, Janecko N, Lim H, et al. Antimicrobial resistance and virulence genes of *Escherichia coli* isolates from swine in Ontario. Appl Environ Microbiol. 2005 Nov; 71 (11):6753–61. https://doi.org/10.1128/AEM.71.11.6753-6761.2005 PMID: 16269706
3. Mulvey MR, Susky E, McCracken M, Morck DW, Read RR. Similar cefoxitin-resistance plasmids circulating in *Escherichia coli* from human and animal sources. Vet Microbiol. 2009 Mar 2; 134(3–4):279–87. https://doi.org/10.1016/j.vetmic.2008.08.018 PMID: 18824313
4. Forward KR, Matheson KM, Hiltz M, Musgrave H, Poppe C. Recovery of cephalosporin-resistant *Escherichia coli* and *Salmonella* from pork, beef and chicken marketed in Nova Scotia. Can J Infect Dis Med Microbiol. 2004 Jul; 15(4):226–30. https://doi.org/10.1155/2004/695305 PMID: 18159497
5. Timonin ME, Poissant J, McLoughlin PD, Hedlin CE, Rubin JE. A survey of the antimicrobial susceptibility of *Escherichia coli* isolated from Sable Island horses. Canadian Journal of Microbiology [Internet]. 2016 Nov 7 [cited 2021 Feb 20]; Available from: https://cdnsciencepub.com/doi/abs/10.1139/cjm-2016-0504 PMID: 28177803
6. de Lagarde M, Fairbrother JM, Arsenault J. Prevalence, Risk Factors, and Characterization of Multidrug Resistant and ESBL/AmpC Producing Escherichia coli in Healthy Horses in Quebec, Canada, in 2015–2016. Animals (Basel) [Internet]. 2020 Mar 20 [cited 2020 Jun 8]; 10(3). Available from: https://www.ncbi.nlm.nih.gov/pmc/articles/PMC7143171/
7. Zhang PLC, Shen X, Chalmers G, Reid-Smith RJ, Slavic D, Dick H, et al. Prevalence and mechanisms of extended-spectrum cephalosporin resistance in clinical and fecal Enterobacteriaceae isolates from dogs in Ontario, Canada. Veterinary Microbiology. 2018 Jan 1; 213:82–8. https://doi.org/10.1016/j.vetmic.2017.11.020 PMID: 29292008
8. Cormier AC, Chalmers G, Cook SR, Zaheer R, Hannon SJ, Booker CW, et al. Presence and Diversity of Extended-Spectrum Cephalosporin Resistance Among *Escherichia coli* from Urban Wastewater and Feedlot Cattle in Alberta, Canada. Microbial Drug Resistance. 2019 Sep 25; 26(3):300–9. https://doi.org/10.1089/mdr.2019.0112 PMID: 31553261
9. Vounba P, Arsenault J, Bada-Alambédji R, Fairbrother JM. Antimicrobial Resistance and Potential Pathogenicity of *Escherichia coli* Isolates from Healthy Broilers in Québec, Canada. Microbial Drug Resistance. 2019 Apr 30; 25(7):1111–21. https://doi.org/10.1089/mdr.2018.0403 PMID: 31038391
10. Chalmers G, Cormier AC, Nadeau M, Côté G, Reid-Smith RJ, Boerlin P. Determinants of virulence and of resistance to ceftiofur, gentamicin, and spectinomycin in clinical *Escherichia coli* from broiler chickens in Québec, Canada. Veterinary Microbiology. 2017 May 1; 203:149–57. https://doi.org/10.1016/j.vetmic.2017.02.005 PMID: 28619137

11. Moffat J, Chalmers G, Reid-Smith R, Mulvey MR, Agunos A, Calvert J, et al. Resistance to extended-spectrum cephalosporins in Escherichia coli and other Enterobacterales from Canadian turkeys. PLoS One. 2020; 15(9):e0236442. https://doi.org/10.1371/journal.pone.0236442 PMID: 32925914

12. Zhang PLC. Resistance to extended-spectrum cephalosporins in Enterobacteriaceae from chickens, dogs, and pigs in Ontario. University of Guelph; 2017.

13. Tassios PT, Gazouli M, Tzelepi E, Milch H, Kozlova N, Sidorenko S, et al. Spread of a *Salmonella* typhimurium clone resistant to expanded-spectrum cephalosporins in three European countries. J Clin Microbiol. 1999 Nov; 37(11):3774–7. https://doi.org/10.1128/JCM.37.11.3774-3777.1999 PMID: 10523600

14. Bauernfeind A, Grimm H, Schweighart S. A new plasmidic cefotaximase in a clinical isolate of *Escherichia coli*. Infection. 1990 Oct; 18(5):294–8. https://doi.org/10.1007/BF01647010 PMID: 2276823

15. Gniadkowski M, Schneider I, Palucha A, Jungwirth R, Mikiewicz B, Bauernfeind A. Cefotaxime-Resistant EnterobacteriaceaeIsolates from a Hospital in Warsaw, Poland: Identification of a New CTX-M-3 Cefotaxime-Hydrolyzing β-Lactamase That Is Closely Related to the CTX-M-1/MEN-1 Enzyme. Antimicrobial Agents and Chemotherapy. 1998 Apr 1; 42(4):827–32. https://doi.org/10.1128/AAC.42.4.827 PMID: 9559791

16. Bauernfeind A, Casellas JM, Goldberg M, Holley M, Jungwirth R, Mangold P, et al. A new plasmidic cefotaximase from patients infected with Salmonella typhimurium. Infection. 1992 Jun; 20(3):158–63. https://doi.org/10.1007/BF01704610 PMID: 1644493

17. Ma L, Ishii Y, Ishiguro M, Matsuzawa H, Yamaguchi K. Cloning and Sequencing of the Gene Encoding Toho-2, a Class A β-Lactamase Preferentially Inhibited by Tazobactam | Antimicrobial Agents and Chemotherapy. Antimicrob Agents Chemother. 1998 May 1; 42(5):1181–6. https://doi.org/10.1128/AAC.42.5.1181 PMID: 9593147

18. Boyd DA, Tyler S, Christianson S, McGeer A, Muller MP, Willey BM, et al. Complete Nucleotide Sequence of a 92-Kilobase Plasmid Harboring the CTX-M-15 Extended-Spectrum Beta-Lactamase Involved in an Outbreak in Long-Term-Care Facilities in Toronto, Canada. Antimicrobial Agents and Chemotherapy. 2004 Oct 1; 48(10):3758–64. https://doi.org/10.1128/AAC.48.10.3758-3764.2004 PMID: 15388431

19. Mulvey MR, Bryce E, Boyd D, Ofner-Agostini M, Christianson S, Simor AE, et al. Ambler Class A Extended-Spectrum Beta-Lactamase-Producing *Escherichia coli* and *Klebsiella spp*. in Canadian Hospitals. Antimicrobial Agents and Chemotherapy. 2004 Apr 1; 48(4):1204–14. https://doi.org/10.1128/AAC.48.4.1204-1214.2004 PMID: 15047521

20. Khashhayar B. Antimicrobial Resistance and Selected β-lactam Resistance Genes in *Escherichia coli* From Canine Urinary Tract Infections. University of Guelph; 2009.

21. Cantón R, González-Alba JM, Galán JC. CTX-M Enzymes: Origin and Diffusion. Front Microbiol [Internet]. 2012 Apr 2 [cited 2021 Apr 19]; 3. Available from: https://www.ncbi.nlm.nih.gov/pmc/articles/PMC3316993/ https://doi.org/10.3389/fmicb.2012.00110 PMID: 22485109

22. Cantón R, Ruiz-Garbajosa P. Co-resistance: an opportunity for the bacteria and resistance genes. Curr Opin Pharmacol. 2011 Oct; 11(5):477–85. https://doi.org/10.1016/j.coph.2011.07.007 PMID: 21840259

23. Mathers AJ, Peirano G, Pitout JDD. The Role of Epidemic Resistance Plasmids and International High-Risk Clones in the Spread of Multidrug-Resistant Enterobacteriaceae. Clinical Microbiology Reviews. 2015 Jul 1; 28(3):565–91. https://doi.org/10.1128/CMR.00116-14 PMID: 25926236

24. Cormier A, Zhang PLC, Chalmers G, Weese JS, Deckert A, Mulvey M, et al. Diversity of CTX-M-positive *Escherichia coli* recovered from animals in Canada. Veterinary Microbiology. 2019 Apr 1; 231:71–5. https://doi.org/10.1016/j.vetmic.2019.02.031 PMID: 30955827

25. Denisuik AJ, Karlowsky JA, Adam HJ, Baxter MR, Lagacé-Wiens PRS, Mulvey MR, et al. Dramatic rise in the proportion of ESBL-producing *Escherichia coli* and *Klebsiella pneumoniae* among clinical isolates identified in Canadian hospital laboratories from 2007 to 2016. Journal of Antimicrobial Chemotherapy. 2019 Aug 1; 74(Supplement_4):iv64–71. https://doi.org/10.1093/jac/dkz289 PMID: 31505647

26. Geser N, Stephan R, Korczak BM, Beutin L, Hächler H. Molecular Identification of Extended-Spectrum-β-Lactamase Genes from Enterobacteriaceae Isolated from Healthy Human Carriers in Switzerland. Antimicrobial Agents and Chemotherapy. 2012 Mar 1; 56(3):1609–12. https://doi.org/10.1128/AAC.05539-11 PMID: 22155836

27. Hall MAL, Dierikx CM, Stuart JC, Voets GM, Munckhof MP van den, Essen-Zandbergen A van, et al. Dutch patients, retail chicken meat and poultry share the same ESBL genes, plasmids and strains. Clinical Microbiology and Infection. 2011; 17(6):873–80. https://doi.org/10.1111/j.1469-0691.2011.03497.x PMID: 21463397

28. Hansen DS, Schumacher H, Hansen F, Stegger M, Hertz FB, Schønning K, et al. Extended-spectrum β-lactamase (ESBL) in Danish clinical isolates of *Escherichia coli* and *Klebsiella pneumoniae*: prevalence, β-lactamase distribution, phylogroups, and co-resistance. Scand J Infect Dis. 2012 Mar; 44(3):174–81. https://doi.org/10.3109/00365548.2011.632642 PMID: 22364227

29. Day MJ, Rodríguez I, van Essen-Zandbergen A, Dierikx C, Kadlec K, Schink A-K, et al. Diversity of STs, plasmids and ESBL genes among *Escherichia coli* from humans, animals and food in Germany, the Netherlands and the UK. Journal of Antimicrobial Chemotherapy. 2016 May 1; 71(5):1178–82. https://doi.org/10.1093/jac/dkv485 PMID: 26803720
30. Irrgang A, Hammerl JA, Falgenhauer L, Guiral E, Schmoger S, Imirzalioglu C, et al. Diversity of CTX-M-1-producing *E. coli* from German food samples and genetic diversity of the *blaCTX-M-1* region on IncI1 ST3 plasmids. Vet Microbiol. 2018 Jul; 221:98–104. https://doi.org/10.1016/j.vetmic.2018.06.003 PMID: 29981716
31. Seiffert SN, Hilty M, Perreten V, Endimiani A. Extended-spectrum cephalosporin-resistant gram-negative organisms in livestock: An emerging problem for human health? Drug Resistance Updates. 2013 Feb 1; 16(1):22–45. https://doi.org/10.1016/j.drup.2012.12.001 PMID: 23395305
32. Mo SS, Telke AA, Osei KO, Sekse C, Slettemeås JS, Urdahl AM, et al. blaCTX–M–1/IncI1-Iγ Plasmids Circulating in *Escherichia coli* From Norwegian Broiler Production Are Related, but Distinguishable. Front Microbiol [Internet]. 2020 Mar 5 [cited 2020 Jun 8]; 11. Available from: https://www.ncbi.nlm.nih.gov/pmc/articles/PMC7066084/ https://doi.org/10.3389/fmicb.2020.00333 PMID: 32194533
33. Zurfluh K, Wang J, Klumpp J, Nüesch-Inderbinen M, Fanning S, Stephan R. Vertical transmission of highly similar blaCTX-M-1-harboring IncI1 plasmids in *Escherichia coli* with different MLST types in the poultry production pyramid. Front Microbiol [Internet]. 2014 Sep 30 [cited 2020 Jun 13]; 5. Available from: https://www.ncbi.nlm.nih.gov/pmc/articles/PMC4179741/
34. Lupo A, Haenni M, Saras E, Gradin J, Madec J-Y, Börjesson S. Is *blaCTX-M-1* Riding the Same Plasmid Among Horses in Sweden and France? Microbial Drug Resistance. 2018 May 24; 24(10):1580–6. https://doi.org/10.1089/mdr.2017.0412 PMID: 29792781
35. Valcek A, Sismova P, Nesporova K, Overballe-Petersen S, Bitar I, Jamborova I, et al. Horsing Around: *Escherichia coli* ST1250 of Equine Origin Harboring Epidemic IncHI1/ST9 Plasmid with *blaCTX-M-1* and an Operon for Short-Chain Fructooligosaccharide Metabolism. Antimicrobial Agents and Chemotherapy [Internet]. 2021 Apr 19 [cited 2021 Apr 23]; 65(5). Available from: https://aac.asm.org/content/65/5/e02556-20
36. Baron S, Le Devendec L, Touzain F, Jouy E, Lucas P, de Boisséson C, et al. Longitudinal study of *Escherichia coli* plasmid resistance to extended-spectrum cephalosporins in free-range broilers. Veterinary Microbiology. 2018 Mar 1; 216:20–4. https://doi.org/10.1016/j.vetmic.2018.01.012 PMID: 29519517
37. Carattoli A, Villa L, Fortini D, García-Fernández A. Contemporary IncI1 plasmids involved in the transmission and spread of antimicrobial resistance in Enterobacteriaceae. Plasmid. 2018 Dec 5;S0147-619X(18)30046-5. https://doi.org/10.1016/j.plasmid.2018.12.001 PMID: 30529488
38. CLSI. Performance Standards for Antimicrobial Disk and Dilution Susceptibility Tests for Bacteria Isolated from Animals. Wayne, Pennsylvania, USA: Clinical and Laboratory Standards Institute; 2015. Report No.: VET01S, 3rd ed.
39. Wick R, Volkening J, Loman N. Porechop [Internet]. 2018. Available from: https://github.com/rrwick/Porechop#full-usage
40. Wick RR, Judd LM, Gorrie CL, Holt KE. Unicycler: Resolving bacterial genome assemblies from short and long sequencing reads. PLOS Computational Biology. 2017 Jun 8; 13(6):e1005595. https://doi.org/10.1371/journal.pcbi.1005595 PMID: 28594827
41. Kolmogorov M, Yuan J, Lin Y, Pevzner PA. Assembly of long, error-prone reads using repeat graphs. Nature Biotechnology. 2019 May; 37(5):540–6. https://doi.org/10.1038/s41587-019-0072-8 PMID: 30936562
42. Walker BJ, Abeel T, Shea T, Priest M, Abouelliel A, Sakthikumar S, et al. Pilon: An Integrated Tool for Comprehensive Microbial Variant Detection and Genome Assembly Improvement. PLOS ONE. 2014 Nov 19; 9(11):e112963. https://doi.org/10.1371/journal.pone.0112963 PMID: 25409509
43. Hunt M, Mather AE, Sánchez-Busó L, Page AJ, Parkhill J, Keane JA, et al. ARIBA: rapid antimicrobial resistance genotyping directly from sequencing reads. Microbial Genomics. 3(10):e000131. https://doi.org/10.1099/mgen.0.000131 PMID: 29177089
44. Seemann T. abricate [Internet]. 2021 [cited 2021 Apr 24]. Available from: https://github.com/tseemann/abricate
45. Zankari E, Hasman H, Cosentino S, Vestergaard M, Rasmussen S, Lund O, et al. Identification of acquired antimicrobial resistance genes. J Antimicrob Chemother. 2012 Nov; 67(11):2640–4. https://doi.org/10.1093/jac/dks261 PMID: 22782487
46. socru: typing of genome-level order and orientation around ribosomal operons in bacteria | Microbiology Society [Internet]. [cited 2021 Apr 21]. Available from: https://www.microbiologyresearch.org/content/journal/mgen/10.1099/mgen.0.000396
47. Seemann T. snippy [Internet]. 2021 [cited 2021 Apr 24]. Available from: https://github.com/tseemann/snippy

48. Clermont O, Dixit OVA, Vangchhia B, Condamine B, Dion S, Bridier-Nahmias A, et al. Characterization and rapid identification of phylogroup G in *Escherichia coli*, a lineage with high virulence and antibiotic resistance potential. Environmental Microbiology. 2019; 21(8):3107–17. https://doi.org/10.1111/1462-2920.14713 PMID: 31188527
49. Waters N. EzClermont: The E. coli Clermont PCR phylotyping tool [Internet]. 2020 [cited 2021 Nov 1]. Available from: https://github.com/nickp60/EzClermont
50. ECTyper (an easy typer) [Internet]. National Microbiology Laboratory; 2021 [cited 2021 Nov 1]. Available from: https://github.com/phac-nml/ecoli_serotyping
51. Sullivan MJ, Petty NK, Beatson SA. Easyfig: a genome comparison visualizer. Bioinformatics. 2011 Apr 1; 27(7):1009–10. https://doi.org/10.1093/bioinformatics/btr039 PMID: 21278367
52. Carattoli A, Zankari E, García-Fernández A, Voldby Larsen M, Lund O, Villa L, et al. In silico detection and typing of plasmids using PlasmidFinder and plasmid multilocus sequence typing. Antimicrob Agents Chemother. 2014 Jul; 58(7):3895–903. https://doi.org/10.1128/AAC.02412-14 PMID: 24777092
53. Xie Z, Tang H. ISEScan: automated identification of insertion sequence elements in prokaryotic genomes. Bioinformatics. 2017 Nov 1; 33(21):3340–7. https://doi.org/10.1093/bioinformatics/btx433 PMID: 29077810
54. Mulvey MR, Soule G, Boyd D, Demczuk W, Ahmed R, the Multi-provincial Salmonella Typhimurium Case Control Study group. Characterization of the First Extended-Spectrum Beta-Lactamase-Producing Salmonella Isolate Identified in Canada. Journal of Clinical Microbiology. 2003 Jan 1; 41(1):460–2. https://doi.org/10.1128/JCM.41.1.460-462.2003 PMID: 12517894
55. Nucleotide BLAST: Search nucleotide databases using a nucleotide query [Internet]. [cited 2021 May 11]. Available from: https://blast.ncbi.nlm.nih.gov/Blast.cgi?PROGRAM=blastn&PAGE_TYPE=BlastSearch&LINK_LOC=blasthome
56. Valcek A, Roer L, Overballe-Petersen S, Hansen F, Bortolaia V, Leekitcharoenphon P, et al. IncI1 ST3 and IncI1 ST7 plasmids from CTX-M-1-producing *Escherichia coli* obtained from patients with bloodstream infections are closely related to plasmids from E. coli of animal origin. J Antimicrob Chemother. 2019 Aug 1; 74(8):2171–5. https://doi.org/10.1093/jac/dkz199 PMID: 31089683
57. Touzain F, Devendec LL, Boisséson C de, Baron S, Jouy E, Perrin-Guyomard A, et al. Characterization of plasmids harboring *blaCTX-M* and *blaCMY* genes in *E. coli* from French broilers. PLOS ONE. 2018 Jan 23; 13(1):e0188768. https://doi.org/10.1371/journal.pone.0188768 PMID: 29360838
58. de Been M, Lanza VF, de Toro M, Scharringa J, Dohmen W, Du Y, et al. Dissemination of cephalosporin resistance genes between Escherichia coli strains from farm animals and humans by specific plasmid lineages. PLoS Genet. 2014 Dec; 10(12):e1004776. https://doi.org/10.1371/journal.pgen.1004776 PMID: 25522320
59. Seemann T. Prokka: rapid prokaryotic genome annotation. Bioinformatics. 2014 Jul 15; 30(14):2068–9. https://doi.org/10.1093/bioinformatics/btu153 PMID: 24642063
60. Page AJ, Cummins CA, Hunt M, Wong VK, Reuter S, Holden MTG, et al. Roary: rapid large-scale prokaryote pan genome analysis. Bioinformatics. 2015 Nov 15; 31(22):3691–3. https://doi.org/10.1093/bioinformatics/btv421 PMID: 26198102
61. Seemann T. snp-dists [Internet]. 2021 [cited 2021 Apr 24]. Available from: https://github.com/tseemann/snp-dists
62. Canada H. Categorization of Antimicrobial Drugs Based on Importance in Human Medicine [Internet]. aem. 2009 [cited 2021 Apr 24]. Available from: https://www.canada.ca/en/health-canada/services/drugs-health-products/veterinary-drugs/antimicrobial-resistance/categorization-antimicrobial-drugs-based-importance-human-medicine.html
63. Antibiotics | Chicken Farmers of Canada [Internet]. 2014 [cited 2021 Apr 24]. Available from: https://www.chickenfarmers.ca/antibiotics/
64. Martin LC, Weir EK, Poppe C, Reid-Smith RJ, Boerlin P. Characterization of blaCMY-2 Plasmids in *Salmonella* and *E. coli* from Food Animals in Canada. Appl Environ Microbiol [Internet]. 2011 Dec 7 [cited 2021 May 10]; Available from: https://aem.asm.org/content/early/2011/12/02/AEM.06498-11 https://doi.org/10.1128/AEM.06498-11 PMID: 22156427
65. Weese JS, Sabino C. Scrutiny of antimicrobial use in racing horses with allergic small airway inflammatory disease. Can Vet J. 2005 May; 46(5):438–9. PMID: 16018565
66. Dolejska M, Villa L, Minoia M, Guardabassi L, Carattoli A. Complete sequences of IncHI1 plasmids carrying *blaCTX-M-1* and qnrS1 in equine *Escherichia coli* provide new insights into plasmid evolution. J Antimicrob Chemother. 2014 Sep; 69(9):2388–93. https://doi.org/10.1093/jac/dku172 PMID: 24862095
67. Fischer EA, Dierikx CM, van Essen-Zandbergen A, van Roermund HJ, Mevius DJ, Stegeman A, et al. The IncI1 plasmid carrying the *blaCTX-M-1* gene persists in in vitro culture of a *Escherichia coli* strain from broilers. BMC Microbiology. 2014 Mar 25; 14(1):77. https://doi.org/10.1186/1471-2180-14-77 PMID: 24666793
